# The Health and Environmental Impact of Plastic Waste Disposal in South African Townships: A Review

**DOI:** 10.3390/ijerph19020779

**Published:** 2022-01-11

**Authors:** Adeleye Ayoade Adeniran, Winston Shakantu

**Affiliations:** Department of Construction Management, School of Built Environment, EBEIT, Nelson Mandela University, Port Elizabeth 6001, South Africa; winston.shakantu@mandela.ac.za

**Keywords:** environmental impact, waste, plastic, recycling, dumping, incineration

## Abstract

Twenty-first century human behaviour continues to escalate activities that result in environmental damage. This calls for environmentally friendly solutions, such as waste recycling and handling, to deal with the increased amount of waste, especially plastics. The plastic materials manufacturing sector is booming, particularly packaging; while only a fraction of its waste is recycled, another fraction is destroyed, and the larger part continues to pollute the environment. In addition to other waste disposal activities, destroying plastic or incineration (which could be for energy recovery) is usually subjected to strict legal requirements because of its effect on the environment. However plastic is destroyed or disposed of, it poses a serious challenge in both the short term and the long term to humans and their natural environment if the process is not efficiently managed. This article describes how a growing amount of plastic waste is disposed of haphazardly in South African townships, while most of the inhabitants are not aware or do not care about the adverse environmental and health effects of these actions. This article examines the environmental and health effects of poor plastic disposal in South African townships as it is in other developing countries to sensitise the citizens to the significance of reducing plastic waste quantities, which will downplay their impact on human health and the environment.

## 1. Introduction

Plastics are chemically created polymers that are used in a broad range of materials, including clothing, medical supplies, water bottles, food packaging, electrical items and construction materials [1]. The last six decades, because of continuous technological progress, have seen a rise in plastics and plastic products manufactured principally from crude oil derivatives because of their relatively low cost of production and “incomparable” usability features [2]. Hence, it is not surprising that researchers have suggested that by 2050, there will be fewer fish than plastics in terms of weight in the oceans [3], and this is informed by the annual estimated 13 million tonnes out of the 500 billion plastic bags used ending up in the ocean and causing the mortality of over 100,000 aquatic beings [4]. South Africa, like the world at large, is experiencing a surge in using plastics, resulting in an ever-increasing amount of plastic waste [5], and plastic waste management, recycling and disposal is a huge concern that places a significant strain on the environment [6]. As a result, it is critical to explore further the consequences of plastic items and waste on human health and the environment, specifically for South African townships.

This article does not cover the complete waste management process, but provides a broad overview of the energy recovery, disposal hazards and negative consequences of plastic waste.

## 2. Literature Review

Plastics, whether thermoplastics or thermosetting polymers, are organic chemicals made up of large molecules primarily made from synthetic oil derivatives, with their quality in terms of impact and fire resistance, flexibility and colouration determined by the many additives incorporated into the raw material [7]. South Africa ranks in the top 20 coastal nations globally in terms of the quantity of mismanaged plastic waste, according to [8], and was thus classified as a substantial donor to the pollution of the marine life by plastics. Recent research contradicts this assertion, however, because of a lack of precise data on waste generation and management worldwide, with [9] indicating that the projected leakage of plastics into the South African aquatic body is suggestively below what [8] estimated. This does not undermine the urgency of addressing the problem in South Africa and elsewhere.

Varnava and Patrickios [10] record that plastic was first used in prehistoric Mesoamerica around 1600 B.C. when natural rubber was polymerised by human hands and fashioned into various useful things. However, when vulcanised rubber and polystyrene (PS) were found in 1839 [11], and the first entirely synthetic polymer, bakelite, was developed in 1907 in Belgium [12], the large-scale manufacturing of plastics and plastic items began. Bakelite was widely used by 1930, particularly in the fashion, communication, automobile and electrical industries, and mass production of plastics which began after another decade, has continued to grow ever since [13].

Global plastic production was assessed to be 245 million tonnes per year in 2008, and accounting for about 40% of total plastic usage in Europe, single-use packaging is currently the largest industry [14]. After this is consumer products (22%) and materials for construction (20%), automotive (9%), electrical (6%) and agriculture applications (3%) [15]. According to estimates from 2015, Asia has the highest plastic production rate of 49% of total global output, with China’s 28% making it the top producer, followed by North America and Europe, with 19% each, and the remainder of the world is insignificant in terms of manufacturing, but not necessarily in terms of plastic use [15].

Around 311 million tonnes of plastics were produced worldwide, which is anticipated to double by 2034 and quadruple by 2050 [16]. Furthermore, according to the International Energy Agency’s World Energy Outlook [17], the largest use of plastic, which is packaging and accounts for 26% of total volume, is expected to continue to grow rapidly, potentially quadrupling in size by 2050, to around 318 million tonnes annually, which is more than the entire contemporary plastic manufacturing.

Plastics are categorised in line with their composition, and the materials used in their production are presented in Table 1 according to types and their properties, health effects and applications [18,19,20,21,22,23,24,25].

**Figure 1:**
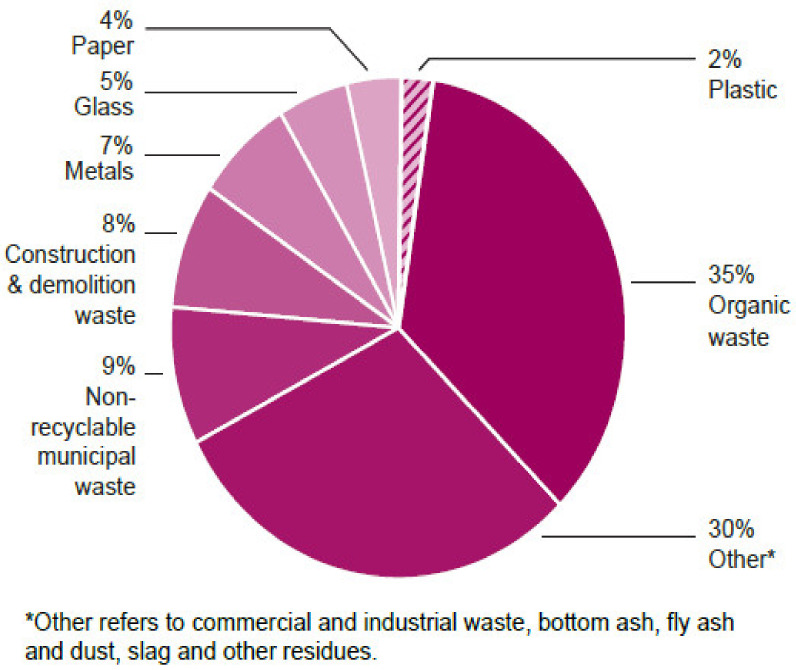
Breakdown of general waste generated in South Africa in 2017.

Approximately 2% of general waste in South Africa is plastic (Figure 1) [26] and mostly ends up in landfills as it is the widespread waste management conventional approach globally; however, the dearth of landfill spaces is becoming a major problem [27,28]. As a result of the types and quantities of harmful compounds present in landfills, as well as their potential for leaching, there is growing public health and environmental concern about landfill effects [29]. Government and other stakeholders worldwide aim to minimise the quantity of waste that is landfilled, but this has been difficult to achieve because approximately 90% of South Africa’s municipal waste is still landfilled [27], compared to 20%, 37% and 60% in Germany, France and England, respectively [30]. Although there is a risk of groundwater and soil contamination from disintegrating plastic additives and leftovers that can linger in the environment for a long time [31], public health and environmental pollution hazards can be mitigated if landfills are effectively managed.

Incineration is a practical alternative to landfilling plastic waste. However, there are increasing concerns about the possibility of hazardous chemicals, such as halogenated additives and polyvinyl chloridebecause the combustion of plastics releases dioxins, furans and polychlorinated biphenyls (PCBs) into the atmosphere during the process [32]. Gilpin et al. [33] highlighted some compounds released during PVC incineration, including acetaldehyde, benzaldehyde, formaldehyde, phosgene, polychlorinated dibenzo-dioxin, hydrochloric acid, propylene and vinyl chloride, among others. Some of the health effects of these compounds include damaging the nervous system, causing lesions, eye and respiratory tract irritation and carcinogenic effects adversely affecting the bone marrow, the liver and the immune system [33]. In addition, the released substances settle on the soil and plants as black carbon, ashes, and many powders, with the potency to drift to the marine environment and when rain falls, some of these toxic composites permeate the soil, pollute the aquifers, or plants cultivated around this soil absorb them and further integrate them into the food chain [34].

Contrary to recycling and landfilling, incineration of plastic is less commonly used for waste management due to its higher immediate possibility for pollution. The only benefit from plastic incineration is the recovery of energy [35] but plastics can be recycled by converting recovered scraps into usable materials. However, since most plastics are non-biodegradable, the best effort will be to reduce waste productions, effectively reuse waste and then recycle [36].

Plastic recycling is an essential part of the global effort toward the reduction of the 8 million tons of plastic debris that empties into the ocean yearly [37], although the plastic recycling terminology is complicated due to its recycling and recovery systems [38]. There are four types of recycling techniques, and the primary technique is one which mechanically reprocesses plastics into an equally strong new product. The next one is called the secondary technique, and it is the mechanical reprocessing of plastics into a product with lesser qualities. The third, which is called the tertiary technique, revolves around the recovery of the chemical elements of the plastics, and the last recovers the chemical constituents of the plastics. Whatever method is used to recycle plastic, the smelting of various sorts of materials often results in phase separation like that of oil and water, and the result is the reason for the structural weakness in the finished product(s), which is accountable for the limited use of these polymer sequences [39]. This is the situation with polypropylene and polyethylene, the two most regularly produced plastics, which has reduced their recycling potential [40]. Despite this, the volume of post-consumer recycled plastics has grown since 1990, albeit it is low in comparison to other products, such as corrugated fiberboard (about 70%) and newspaper (nearly 80%) [41]. As documented by Geyer et al. [42] in 2015, about 9% of the world’s 6.3 billion tonnes of plastic garbage were recycled, while the other 12% and 79% were burnt and landfilled, respectively. The global rate of recycling in 2016, however, increased to over 14% of total plastic waste [43], with countries such as Japan, a major contributor, increasing waste recycling from 39% in 1996 to 83% in 2014 [44,45]. 

## 3. Materials and Methods

Several resource materials and databases, including ACS Publications Web of Science, Cambridge Journal, Government Policy Documents, Google Scholar, ProQuest Ebook Central, ScienceDirect and Springer Link, were used to assess important information for this current review. “Plastic waste recycling”, “challenges related with plastic waste”, “health concerns associated with plastic waste”, “recycled plastic”, and “environmental benefits of plastic recycling” were keywords used to find relevant articles.

Any item that contained the phrases “plastic waste” or “recycled plastic” was considered related during the search. Most notably, the challenges associated with plastic trash internationally and specifically in South Africa, as well as health and environmental issues related to plastic garbage and mitigation methods, were closely examined. From the resources available, a large volume of literature was gathered, including articles from peer-reviewed journals, conference proceedings, PhD theses and white papers. However, papers with very limited relevance to the topic were excluded from the study, and some bounding conditions used for screening of relevant papers were:

- only articles published in peer-reviewed journals, peer-reviewed conference proceedings and theses were considered
- the relevant documents considered in this study were from 2010 to 2021.
- papers addressing only the environmental and health impact of plastic waste disposal were considered.

The study included all articles that met these three criteria, and the screening process yielded 71 peer-reviewed papers.

## 4. Status Quo of South African Townships

**Figure 2:**
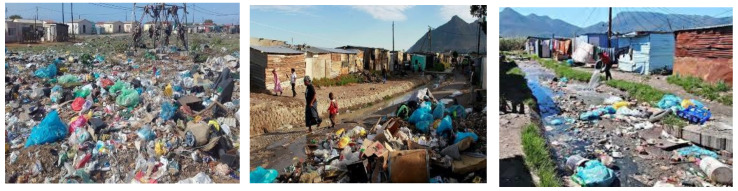
Random pictures of waste around townships in Port Elizabeth, Durban and Johannesburg.

In South Africa, the terminology “township” or “location” usually relates to the largely underdeveloped racially divided metropolitan areas that were earmarked for non-whites, primarily Indians, Africans, and people of colour, from the late 19th century until the fall of apartheid, and they were typically constructed on the outskirts of towns and cities [45]. As of 2007, a quarter of South Africa’s population was indicated to be living in the 76 largest townships [46]. Overcrowded communities in developing cities, despite being linked to municipal water supply, struggle with constant water delivery due to ageing infrastructure, which results in inconsistent availability, and the community in these situations, relies on unofficial water sources to survive [27] that are typically polluted rivers which are laden with heavy metals, diseases, and organic waste [46]. The average number of persons living in shacks is 3.1, with 28% of shack family units experiencing congestion, while a household is considered over-crowded if there are more than two people per room [27]. Because of the poor level of official waste management in these locations [47] (see Figure 2), the communities are at risk of water and/or airborne ailments [48]. In addition, as the world’s population grows, so does the demand for water [49]. The wealthier the economy and the greater the proportion of people living in townships, the more waste and pollution is produced [50]. As a result, waste creation is inevitable, and finding environmentally friendly, economically viable, and socially beneficial waste management methods and technologies has become a major source of concern [51]. Several stakeholders perform a vital role toward ensuring that the current waste management systems succeed, but according to the Constitution of South Africa (Act 108 of 1996) and the Municipal Systems Act, 2000, the municipalities are delegated the responsibility of waste collection, waste disposal and waste cleaning services to all its residents [52].

## 5. The Environmental and Health Impact of Plastic Waste Disposal in South African Townships

This section discusses the environmental and health impact of plastic waste disposal in South African townships as retrieved from secondary sources.

### 5.1. Environmental Impact

The increased human population impacts the distribution of plastic waste, and this can ultimately lead to pollution of the environment, which is evident in the decline of the natural environment [53], mortality of aquatic organisms [54] and blockage of sewage systems, especially in third world countries [55], thereby resulting in breeding grounds for mosquitoes and other disease-causing vectors as well as foul odours [56], reduced aeration and water percolation, causing reduced productivity in agricultural lands [57,58].

In Lekwa Local Municipality, for example, [59] identified human exposure to the identified hazards through the air, water, and soil through dermal, oral and skin routes as high environmental health risk potential to the local communities. Also, in Fisantekraal and other South African townships, [60] stated that it is impracticable to regulate the volume of plastic waste dispersed by the wind across and into the river, which in turn harms the animals and the river. Furthermore, [61] indicated that plastic waste mismanagement has recently become prevalent in some eThekwini municipality townships and is evident in most South African townships.

In the Tyume River, [62] observed that plastics are the common floating debris materials and the most pervasive form of waste along this waterway as well as its surrounding environment, hanging on trees and littering open spaces, presenting a threat to humans, animals and aquatic life. Plastic additives can leach and ultimately permeate into diverse elements of the environment, causing water and soil pollution. Dumping or landfilling plastics causes biotic and abiotic plastic degradation, and the resultant microplastics [63] and synthetic polymer fibres [64] have been discovered to remain evident five years after their application to soil and sewage sludge.

### 5.2. Health Impact

Plastic polymers are commonly assumed to be harmless, yet they pose little risk to society; however, diverse types of additives, as well as residual monomers presumably preserved from these polymers, are speculated to be the source of the health risks. [45]. Most of the plastic additives are known endocrine disruptors and carcinogens [65], and these chemicals harm humans primarily through skin contact (linked to dermatitis), ingestion and inhalation [66]. Microplastics are vital toxins that can form complexes in the food chain after being consumed by a variety of marine and freshwater life, resulting in a variety of health problems [67]. When and if consumed, animals exposed to plastic additives and microplastics can be harmful to humans, and through the detection of environmental contaminants, biomonitoring investigations on human tissues have indicated that plastic elements are found in the human species [68]. All the above are not just localised but global issues and hence applicable to South African townships.

Between 1994 and 2009, consumption of fish in South Africa increased by over 26%, posing human health risks because the ingestion of some aquatic species might trigger the transmission of microplastics, toxins and microorganisms to humans [69]. Ref. [70] research conducted for the World Health Organization (WHO) regarding the risk of microplastics to humans showed that there is inadequate data to draw firm conclusions on nanoparticle toxicity as no reliable evidence suggests it is a concern. However, subject to the dependence of the population on seafood, a varied amount of plastics has been quantified to have been ingested from such food groups as mussels and shrimp in fifty countries in Europe, the Persian Gulf and China [71]. These food groups are taken whole, unlike fish, which is normally cleaned first to eliminate microplastics from the digestive system. For South Africa, edible marine organisms that have been researched for microplastics are brown mussels [72] and four species of estuarial fish [73], but data on levels of transferal of microplastics from edible aquatic species to humans were unavailable for South Africa. [74] discovered that people who ingest a variety of marine species, including filter feeders(e.g., mussels and oysters), may be exposed to microplastics.

Microplastics have been found in human faeces in several investigations, with most particles (90 per cent) being expelled [75]. Some particles may go from the stomach to the lymphatic and circulatory systems, whereas others are more likely to pass through cell tissues, the blood-brain barrier and the placenta [76]. The human body reacts to these particles by triggering immunosuppression, immunological activation and aberrant inflammatory responses [77]. Unfortunately, there is a void in the literature in South Africa at the time of this study, but considering the level of dietary seafood content of a significant section of the country’s population, this area should be prioritised for future research. In humans, however, air inhalation and drinking water appear to be the primary pathways of microplastic uptake, with ingestion as a secondary route [70]. Waste (including plastic) burning is identified in townships by Mngomezulu [50], Nkosi [78] and Muchapondwa [79], and the particles produced by this can be inhaled or ingested when they settle on the water. Microplastics are classified as harmful vectors because they may enable chemical transfer in food types consumed by humans; hence, the chemical ingestion related to microplastics may be a greater concern than the intake of the plastics themselves.

## 6. Recommendations

With the ongoing concerns over global warming, countries are working to reduce environmental and health challenges caused by plastic waste by lowering the manufacture of plastics and plastic products, prohibiting excessive packaging, litter collection and recycling. In contributing to this, this paper recommends the need to enforce realistic policies. It is also essential for the government to implement and enforce regulations that will check processes of manufacturing, consuming, using and the final disposal of plastics. To prevent zero diversion to landfills and indiscriminate disposal to the environment, the 3Rs, reduce, reuse, and recycle must be employed at all stages. Waste management plays a major role in reducing the toxic effects of plastic wastes on public health and the environment, and hence there is a necessity for the advancement of practices that will ensure proper plastic waste collection, treatment and disposal.

Furthermore, the general populace must be educated on the potential health and environmental effects of plastic waste pollution as this will help towards reducing the pollution rate and conserve the quality of the natural environment. Also, bioplastics is a plastic produced from cellulose that is made of wood pulp by a British chemist in the 1850s, and if manufacturers can embrace its use, the problem of plastic waste generation and the accompanying environmental and public health effects can be handled. Furthermore, biodegradability with little or no toxic residue will also aid in protecting the ecosystem from the dangers of traditional plastic wastes.

## 7. Conclusions

Plastic wastes have been acknowledged as a serious environmental concern by various studies on global plastic manufacturing and the resulting waste pollution. The effect of plastic waste on the environment and its inhabitants is a basis of public concern, necessitating saving ecosystems. While plastics are beneficial in daily living, the harmful elements from which they are made must be strictly regulated to ensure the health and environmental safety. Reducing human exposure to emitted toxicants from plastic wastes boosts the prospect of a healthy society and a clean environment.

Health agencies and government organisations must enact and implement environmental rules that will track plastic manufacturing, usage, and disposal. Additionally, some dangerous chemical components utilised in the production of plastics should be abolished in consumer goods and plastic products that interact directly with liquids and foods.

## Figures and Tables

**Table 1:** Types of plastics, their properties and common uses.

Symbols	Types of Plastics	Common Uses	Properties	Negative Health Effect	Recycled Into
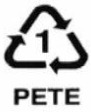	Polyethylene terephthalates	Water bottles, soft drinks, salad dressing and domes, containers and biscuit trays	Tough, clear, solvent resistant, a barrier to moisture and gas softens at 80 °C	Causes carcinogens, vomiting, diarrhoea [19]	Sleeping bag and pillow filling, carpeting, clothing, soft drink bottles, building insulation
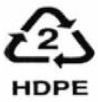	High-density polyethylene (HDPE)	Freezer and shopping bags, buckets, shampoo, ice cream and milk containers, juice bottles, chemical and detergent bottles, rigid agricultural pipe, crates	Hard to semi-flexible, resistant to chemicals and moisture, waxy surface, opaque, softens at 75 °C, easily coloured, processed and formed	Stomach ulcers [20]	Recycling bins, compost bins,
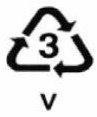	i. Polyvinyl chloride (PVC) ii. Plasticized polyvinyl chloride PVC-P	Cosmetic containers, plumbing pipes and fittings, electrical conduct, blister packs, wall cladding, roof sheeting, bottles, garden hose, shoe soles, cable sheathing, blood bags and tubing	i. Strong, tough, softens at 80 °C, can be solvent welded and clear. ii. Flexible, clear, elastic, can be solvent welded	Interferes with hormonal development [21]	Compost bins
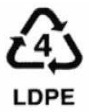	Low-density polyethylene (LDPE)	Refuse bags, irrigation tubings, mulch film, cling wrap, garbage bags, squeeze bottles	Soft flexible, waxy surface, translucent, softens at 70 °C, scratches easily	Not recyclable [22]	Bin liners, pallet sheets
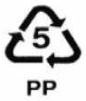	Polypropylene (PP)	Lunch boxes, microwave dishes, garden furniture, kettles, bottles and ice cream tubs, potato chip bags, straws and packaging tape	Hard and translucent, softens at 140 °C, withstands solvents, versatile	No known effects [23]	Pegs, bins, pipes, pallet sheets
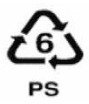	i. Polysterene (PS) ii. Expanded polysterene (PS)	CD cases, plastic cutlery, imitation glassware, low-cost brittle toys, video cases/foamed polystyrene cups, protective packaging, building and food insulation	i. Semi-tough glassy rigid clear or opaque, material, it softens at 95 °C, affected by fat, acids and solvents, but resistant to salt solutions and alkalis, low water absorption, when not pigmented it is clear, odour- and taste-free. ii. Special types PS are available for special applications	Takes a thousand years to degrade [24]	Recycle bins
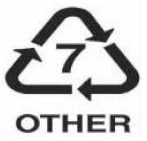	Polycarbonate and others	Automotive and appliance components, computers, electronics, cooler bottles, packaging	Includes all resins and multi-materials (e.g., laminates) properties dependent on plastic or combination of plastics	Obesity, cancer, endocrine problems in foetuses and children [25]	Recycle bins
